# The Apelin/APJ System in Psychosis and Neuropathy

**DOI:** 10.3389/fphar.2020.00320

**Published:** 2020-03-13

**Authors:** Shuang-Yu Lv, Wei-Dong Chen, Yan-Dong Wang

**Affiliations:** ^1^ Key Laboratory of Receptors-Mediated Gene Regulation and Drug Discovery, School of Medicine, Henan University, Kaifeng, China; ^2^ Key Laboratory of Molecular Pathology, School of Basic Medical Science, Inner Mongolia Medical University, Hohhot, China; ^3^ State Key Laboratory of Chemical Resource Engineering, College of Life Science and Technology, Beijing University of Chemical Technology, Beijing, China

**Keywords:** apelin, pain, memory, brain injury, neuroprotection

## Abstract

Apelin, an endogenous neuropeptide, has been identified as the cognate ligand for the G-protein-coupled receptor APJ. Apelin, APJ messenger RNA, and protein are widely expressed in the central nervous system and peripheral tissues of humans and animals. The apelin/APJ system has been implicated in diverse physiological and pathological processes. The present article reviews the progress of the latest research investigating the apelin/APJ system in pain, depression, anxiety, memory, epilepsy, neuroprotection, stroke, and brain injury and protection, and highlights its promising potential as a therapeutic target for treatment of psychosis and neuropathy.

## Introduction

Apelin (APLN), an endogenous ligand of the APJ receptor (APLNR), was first isolated from bovine stomach tissue (Tatemoto et al., 1998). The precursor of apelin, preproapelin, contains 77 amino acids, and undergoes enzymolysis and process into various derivative molecular forms in different tissues, including apelin-36, apelin-26, apelin-19, apelin-17, apelin-13, Pyr-apelin-13, and apelin-12 (Tatemoto et al., 1998; Kawamata et al., 2001; Shin et al., 2018). Pyr-apelin-13 is the N-terminally pyroglutamate-modified apelin-13, and this modified isoform\ has increased stability, as reflected in increased plasma half-life (Zhen et al., 2013). Apelin-55 and apelin-36 can be processed by proprotein convertase subtilisin kexin type 3 (PCSK3) (Shin et al., 2013). The post-translational modifications can occur by angiotensin converting enzyme-2 (ACE2), which removes the C-terminal phenylalanine of all apelin isoforms (Vickers et al., 2002). The shorter forms of apelin, such as apelin-13 and apelin-17, exert more potent effects than the longer forms (Tatemoto et al., 1998; Hosoya et al., 2000; Kawamata et al., 2001). The putative receptor protein related to angiotensin II receptor type-1 (AT1R), known as APJ, is a G-protein-coupled receptor comprising 380 amino acids in human (O'Dowd et al., 1993).

Recently, a novel endogenous ligand for APJ receptor, named Apela/Elabela/Toddler, was identified (Chng et al., 2013; Pauli et al., 2014). Human *elabela* was comprised of three exons on chromosome 4. The *elabela* encodes a conserved 54-amino acids protein, containing an N-terminal signal-peptide and a mature 32- amino acids peptide, named Elabela (Chng et al., 2013). The human *elabela* transcripts have been found in embryonic stem cells, induced pluripotent stem cells, kidney, heart, and blood vessels (Schreiber et al., 2016). Many biological functions of Elabela has been emerged in both embryos and adult organisms, such as dysontogenesis, self-renewing of human embryonic stem cells, endoderm differentiation (Xu et al., 2018).

In humans, the highest levels of *aplnr* mRNA in the CNS are found in the spinal cord, corpus callosum and medulla, while the highest levels in the periphery are found in the spleen and placenta (Edinger et al., 1998; Medhurst et al., 2003). APLNR protein has been found in human cardiomyocytes, vascular endothelial cells, and smooth muscle cells (Kleinz and Davenport, 2005). The distribution of APLNR protein in the human brain, however, remains unclear. Similar to APJ, *apln* mRNA and APLN peptide are widely distributed in the CNS and periphery, and there is a large amount of overlap in the expression profiles of transcripts and protein (Pitkin et al., 2010). As showed in **Supplementary Figure 1**, date from Allen Human Brain Atlas indicates that the high expression of human *apln* and *aplnr* gene were found in several brain regions, including cerebral nuclei, hypothalamus, thalamus, midbrain tegmentum, pons, gracile nucleus, and spinal trigeminal nucleus (Hawrylycz et al., 2012). The detection for the protein expression of APLN and APLNR in CNS also were done using immunoactivity detection. The results need to be confirmed using mass spectrometry in the near future. Whether the central human and rodent apelinergic system gene/protein expression is conserved is still not clear (Tatemoto et al., 1998).

The apelin/APJ system is involved in a variety of physiological functions and pathological processes, including cardiovascular disease, angiogenesis, energy metabolism, and fluid homeostasis (Chapman et al., 2014). Multiple publications indicate that apelin may play an essential role in CNS diseases (Dai et al., 2013). This article provides an overview of the latest advances in the understanding of the signaling pathways and physiological and pathophysiological role of apelin/APJ in pain, depression, anxiety, memory, epilepsy, neuroprotection, stroke, brain injury, and protection.

### Pain

Apelin/APJ system produces a dual function in pain, including acute pain, inflammatory pain, and neuropathic pain. Intracerebroventricular (i.c.v., 0.3–3 µg/mouse) or intrathecal (i.t., 0.3–3 nmol/mouse) administration of apelin-13 resulted in a marked antinociception in the mouse tail-flick test (Xu et al., 2009; Lv et al., 2013). In the mouse writhing test, apelin-13 (i.c.v., 0.3–3 µg/mouse) induced an inhibitory effect on the number of writhes, and this effect was reversed by apelin-13(F13A) and β-funaltrexamine hydrochloride, indicating that the antinociception was mediated by APJ and the µ-opioid receptor (Lv et al., 2012b). It was reported that the human APJ formed a heterodimer with κ opioid receptor (KOR), which imply that APJ/KOR may be a potential target for the development of therapeutic medicines for cerebrovascular and cardiovascular diseases. (Li et al., 2012). In addition, the APJ was activated through coupling to G_q/11_ stimulating phospholipase C beta (PLC-β) signaling (Hosoya et al., 2000) and coupling to G_i/o_ stimulating mitogen-activated protein kinase (MAPK) cascade *via* protein kinase C (PKC) (Szokodi et al., 2002; O'Carroll et al., 2013).

Recently, Turtay et al. reported that intraperitoneal (i.p.) injection of apelin-13 (100 µg/kg) exerted an analgesic effect in both the hot-plate and the tail-flick tests in rats, and that antinociception was reduced by ondansetron (Turtay et al., 2015). Chronic apelin-13 (3 µg/rat) injection resulted in tolerance to its antinociceptive effect and a decrease in APLNR protein expression in the lumbar spinal cord (Abbasloo et al., 2016).

The apelin/APJ system plays a role in chronic (neuropathic) and acute pain. Chronic i.t. injection of Pyr-apelin-13 (1 and 5 µg/rat) attenuated neuropathic pain and reduced caspase-3 levels in rat spinal cord tissues (Hajimashhadi et al., 2017). The spinal cord of rats with chronic constriction injury (CCI) exhibited higher levels of *apln* and *aplnr* mRNA, and APLN and APLNR protein than vehicle control, and apelin-13 (i.t., 10 µg/rat) exerted no effect on the neuropathic nociceptive response (Xiong et al., 2017). However, the APJ antagonist ML221 reduced CCI-induced pain hypersensitivity, and inhibited phosphorylated extracellular signal-related kinase (ERK) in the spinal dorsal horn (Xiong et al., 2017).

Moreover, apelin has been shown to cause hyperalgesia under some conditions. Chen et al. reported that apelin (i.c.v., 0.4 µmol/rat) decreased pain threshold in the rat tail-flick test (Chen and Bai, 2008). In the formalin test, i.t. administration of 3 nmol/mouse apelin-13 induced hyperalgesia, and this process was related to APJ and the gamma-aminobutyric acid receptor type A (GABAA) receptor (Lv et al., 2013). Peripheral injection with apelin-13 (100  and 300 mg/kg) increased pain sensitivity in a mouse model of thermal stimuli-induced acute pain (Canpolat et al., 2016). Additionally, apelin, tumor necrosis factor-alpha (TNF-α), and interleukin (IL)-6 may be involved in the therapeutic effect of electroacupuncture (EA) on knee osteoarthritis, a common cause of joint pain (Ju et al., 2015). In a rat model of complete Freund's adjuvant (CFA)-induced inflammatory pain, EA treatment alleviated CFA-induced decrease in *apln*/*aplnr* mRNA and APLN/APLNR protein expression in the spinal cord, suggesting that EA stimulation could inhibit inflammatory pain, in part, by restoring *apln*/*aplnr* mRNA and APLN/APLNR protein (Wang et al., 2016).

These inconsistent results of the apelin on pain regulation are difﬁcult to explain. It may be due to the different kind of animal model of pain, doses, animal species, administration routes, time of injection, forms of apelin, etc. The main molecular mechanism of apelin/APJ on pain was related to opioid receptor, GABA receptor, and ERK pathway. The effect of apelin/APJ in pain animal models had been extensively studied. However, the roles in primary afferent inputs, pain modulation at the spinal level, and plasticity after nerve injury or inflammation remain unclear. The apelin/APJ systems may be developed as novel analgesics.

### Depression and Anxiety

Apelin exhibited a double-edged sword effect in animal models of depression and an anxiolytic effect in animal models of anxiety. Numerous neuropeptides have been shown to be affected by stress or to be involved in stress response in various animal models (Kormos and Gaszner, 2013). *aplnr* mRNA has been found in the amygdala, hypothalamus, Ammon's horn, and the dentate gyrus (Lee et al., 2000; Reaux et al., 2001), suggesting a potential role of the apelin/APJ system in emotional behavior. Peritoneal dialysis patients with depression and anxiety had a significantly higher serum apelin than those without depression and anxiety (Oguz et al., 2016).

Dysregulation of the hypothalamic-pituitary-adrenal (HPA) axis has been observed in depressed patients (Gillespie and Nemeroff, 2005). Persistent enhancement of stress reactivity heightened HPA axis activity in some depressed patients (Shelton, 2007). Newson et al. indicate that APJ has a role in regulation of the HPA axis in response to some acute stressors (Newson et al., 2013). Chronic i.c.v. infusion of 2 µg apelin-13 upregulated the brain-derived neurotrophic factor (BDNF) against chronic stress-induced depression-like phenotypes by ameliorating HPA axis and hippocampal glucocorticoid receptor dysfunctions (Dai et al., 2018). The role of apelin in depression, however, is controversial. Lv et al. reported that apelin-13 (i.c.v., 0.3–3 µg/mouse) prolonged immobility time in the both forced swim and tail suspension tests, indicating that central apelin-13 promoted depression (Lv et al., 2012a). Repeated injection of apelin-13 (2 µg/rat/d) produced an antidepressant effect in the rat forced swim test, and the PI3K and ERK signaling pathways are involved in this process (Li et al., 2016). Xiao et al. found that intrahippocampal administration of apelin-13 (1–4 µg/rat) produced an antidepressant effect in the rat forced swim test (Xiao et al., 2018).

Telegdy et al. reported that apelin-13 (i.c.v., 0.5 µg/mouse) exhibited an anxiolytic effect in the elevated plus maze, and the antianxiety of apelin-13 was mediated by α-adrenergic, β-adrenergic, dopaminergic, and 5-HT_2_ serotonergic receptors (Telegdy and Jaszberenyi, 2014). Chronic i.p. injection of apelin-13 (20 nmol/kg/d) alleviated anxiety-like behavior induced by chronic normobaric hypoxia in mice (Fan et al., 2017). This effect was mediated by suppressing nuclear factor κB (NF-κB) activation in the microglia of the hippocampus (Fan et al., 2017). Additionally, peripheral injection of apelin-13 in mice with chronic normobaric hypoxia reversed the reduction of silent mating type information regulation 2 homolog 1 (SIRT1) expression in the hippocampus (Fan et al., 2018). Apelin-13 ameliorated the anxiety-like behavior induced by chronic normobaric hypoxia, which was antagonized by the SIRT1 inhibitor EX-527 (Fan et al., 2018 The result indicated that SIRT1 was involved in the anxiolytic activity of apelin-13 in the chronic normobaric hypoxia model by suppressing the NF-κB pathway.

The different effects of apelin on depression, however, may be due to different injection methods and/or different animal species. Moreover, It was reported that the forced swim test does not reﬂect depression (Molendijk and de Kloet, 2015), which may explain the different phenomenon of apelin-treated animals. In addition, the research about apelinergic system is restricted to rodent depression models. The clinical research should been performed to evaluate the effects of apelin in human.

### Memory and Epilepsy

Central apelin has a regulatory effect on memory and epilepsy, and it could protective memory impairment in rodents. *apln*/*aplnr* mRNA and APLN/APLNR protein have been found in the hippocampus, amygdala, and cerebral cortex (Hosoya et al., 2000; Lee et al., 2000; Medhurst et al., 2003), areas known to be closely related to learning and memory. This indicates that the apelin/APJ system may potentially play a role in regulating memory processes. Apelin-13 (i.c.v., 2 µg/mouse) improved memory consolidation in a passive avoidance paradigm in mice, and several neurotransmitters, including α-adrenaline, serotonin, choline, dopamine, GABA, and nitric oxide, were involved in the process (Telegdy et al., 2013). Repeated i.c.v. treatment with apelin-13 (2 µg/rat/d) ameliorated memory impairment in rats induced by exposure to the forced swim stress using the novel object recognition test, and this action was mediated by the PI3K and ERK1/2 pathways (Li et al., 2016). However, other reports have shown that apelin plays an opposite role in learning and memory. Apelin-13 (i.c.v., 1 nmol/mouse) impaired the formation-but not the acquisition-of short-term memory, and blocked consolidation, but not acquisition and recall, of long-term memory in a novel object recognition task (Han et al., 2014). The timing of apelin injection into brain regions may be an important factor to explain the inconsistent role of apelin on acquisition, consolidation or recall of object memory. Han et al. showed that i.c.v. apelin blocked fear acquisition but not fear consolidation or expression in fear memory of rats (Han et al., 2016). In a rat model of 6-hydroxydopamine (OHDA)-induced parkinsonism, apelin-13 (1, 2, and 3 µg/rat) injected into the substantia nigra significantly reduced the increase in escape latency and distance traveled in the Morris water maze test, and the decrease in exploration index in novel object recognition and object location tasks (Haghparast et al., 2018). The different effects of apelin on learn and memory may be attributed to the treatment methods, doses, animal species, and memory models, *ect*. The role apelin/APJ on memory is complicated, further study should be performed to confirm its effect using the *apln* or *aplnr* transgenic animal.

The level of apelin expression in the temporal neocortex of patients with temporal lobe epilepsy was remarkably higher than that in control patients (Zhang et al., 2011). APLN protein in the hippocampus and adjacent cortex was markedly up-regulated in an epileptic rat model compared with control (Zhang et al., 2011). These results demonstrate that apelin may be involved in the pathogenesis of epilepsy.

### Stroke and Neuroprotection

Apelin ameliorated stroke and had neuroprotective effect by anti-apoptosis. In cultured mouse cortical neurons, apelin-13 (0.5, 5 nmol/L) prevented neuronal apoptosis by suppressing the generation of reactive oxygen species, cytochrome c release, mitochondria membrane depolarization, and caspase-3 activity (Zeng et al., 2010). Intravitreal injection with apelin-36 (0.33 nmol/eye) ameliorated NMDA-induced ganglion cell death in mouse retina *in vivo*. This function was independent of the APJ receptor and apelin-36 can directly act on NMDA receptors and/or antagonize the binding of NMDA on NMDA receptors (Sakamoto et al., 2016).

Ischemic stroke is a common neurological disease, and generally leads to brain damage and neuronal cell death (Park et al., 2017). Chen et al. showed that intranasal delivery of apelin-13 (4 mg/kg) reduced infarct volume and neuron death in the penumbra of ischemic stroke mice (Chen et al., 2015). Apelin-13 produced neuroptotective effect by suppressing gene expression of the inflammatory cytokines, such as IL-1β, TNF-α, and intercellular adhesion molecule (ICAM)-1 (Xin et al., 2015). Central apelin-13 (100 µg/kg) demonstrated a role in anti-apoptosis, including a reduction in the number of apoptotic cells, inhibition of Bax and cleaved-caspase3, and stimulation of Bcl2 in ischemic stroke (Yang et al., 2016). The mechanism of this action involved the activation of the AMPK signaling pathway (Yang et al., 2016). In addition, it was showed that apelin could reduce the motor neuron apoptosis in the spinal cord anterior horn and delay the onset of apoptosis (Li et al., 2011). The neuroprotection conferred by apelin-13 (50 µg/kg) on ischemia/reperfusion (I/R) injury involved differentially expressed microRNAs and their target genes, and the predicted targets of microRNAs were related to the MAPK or JAK-ATAT signaling pathways (Wang et al., 2018).

Recently, clinical studies have been focused on the genetic relationship between the *aplnr* variant and ischemic stroke. The rs9943582 variant of *aplnr* was associated with a significantly higher risk for brain infarction in the Japanese population (Hata et al., 2007). In contrast, Zhang et al. found that the *aplnr* variant rs9943582 had no relationship with age at onset and clinical outcomes of ischemic stroke in Chinese patients (Zhang et al., 2017). Wang et al. reported that there was no allelic or genotypic association between rs9943582 and ischemic stroke in the Chinese Han GeneID population (Wang et al., 2017). These conflicting results may be due to the different genetic characteristics of Chinese and Japanese populations, or different sample sizes, methodological and statistical methods. The previous report indicates that the effect of apelin-36 (0.5 µg/rat) on infarct and apoptosis caused by I/R injury was mediated by inhibition of the endoplasmic reticulum stress/unfolded protein response (ERS/UPR) activation (Qiu et al., 2017). The protection conferred by apelin-13 on cerebral I/R injury-induced neuronal apoptosis was through the activation of Gαi/Gαq-CK2 signaling (Wu et al., 2018).

All the above studies indicate that apelin could alleviate stroke and exhibit a neuroprotective effect *via* inhibiting neuronal apoptosis, which was mediated by AMPK/Bax/cleaved-caspase3/Bcl2, ERS/UPR, and/or Gαi/Gαq-CK2 pathways. More clinical research is wanted for an improved understanding of the apelin/APJ system in stroke and for the application of apelin in clinical practice for the patients with stroke.

### Brain Injury and Protection

Supraspinal administration of apelin mitigated the brain injury and showed a neuroprotective role in animal models. Apelin-13 (i.c.v., 50, 100 µg/rat, Khaksari et al.; 20 µg/rat, Yan et al., 2015) mitigated cerebral damage in rats induced by transient focal cerebral ischemia *via* inhibition of apoptosis and caspase-3 activation (Khaksari et al., 2012; Yan et al., 2015). Apelin-13 (i.c.v., 50, 100 µg/kg) ameliorated I/R injury in the mouse brain by activating the PI3K/Akt and ERK1/2 signaling pathways (Yang et al., 2014). Apelin-13 (i.c.v., 50 µg/kg) alleviated damage to the mouse blood-brain barrier from ischemic injury *via* improvement of aquaporin-4 (AQP4), and the increase of AQP4 caused by apelin-13 was mediated through the PI3K/Akt and ERK pathways (Chu et al., 2016). I.c.v. administration of 50 µg/mouse apelin-13 alleviated mouse brain damage induced by traumatic brain injury *via* the suppression of autophagy (Bao et al., 2015).

The studies indicate that central apelin could ameliorate brain injury and induce a neuroprotective effect, which was mediated by PI3K/Akt/ERK, AQP4, and/or inhibiting autophagy and apoptotic pathways. Autophagy and apoptosis are two major physiologic processes to maintain the cellular homeostasis. We infer that apelin may be a potential regulatory factor in cell physiology and neurodegenerative disorders. It is necessary to ascertain whether apelin could pass blood brain barrier and whether the protective effect is still effective through peripheral treatment, such as intravenous injection.

## Conclusion

**Figure 1:**
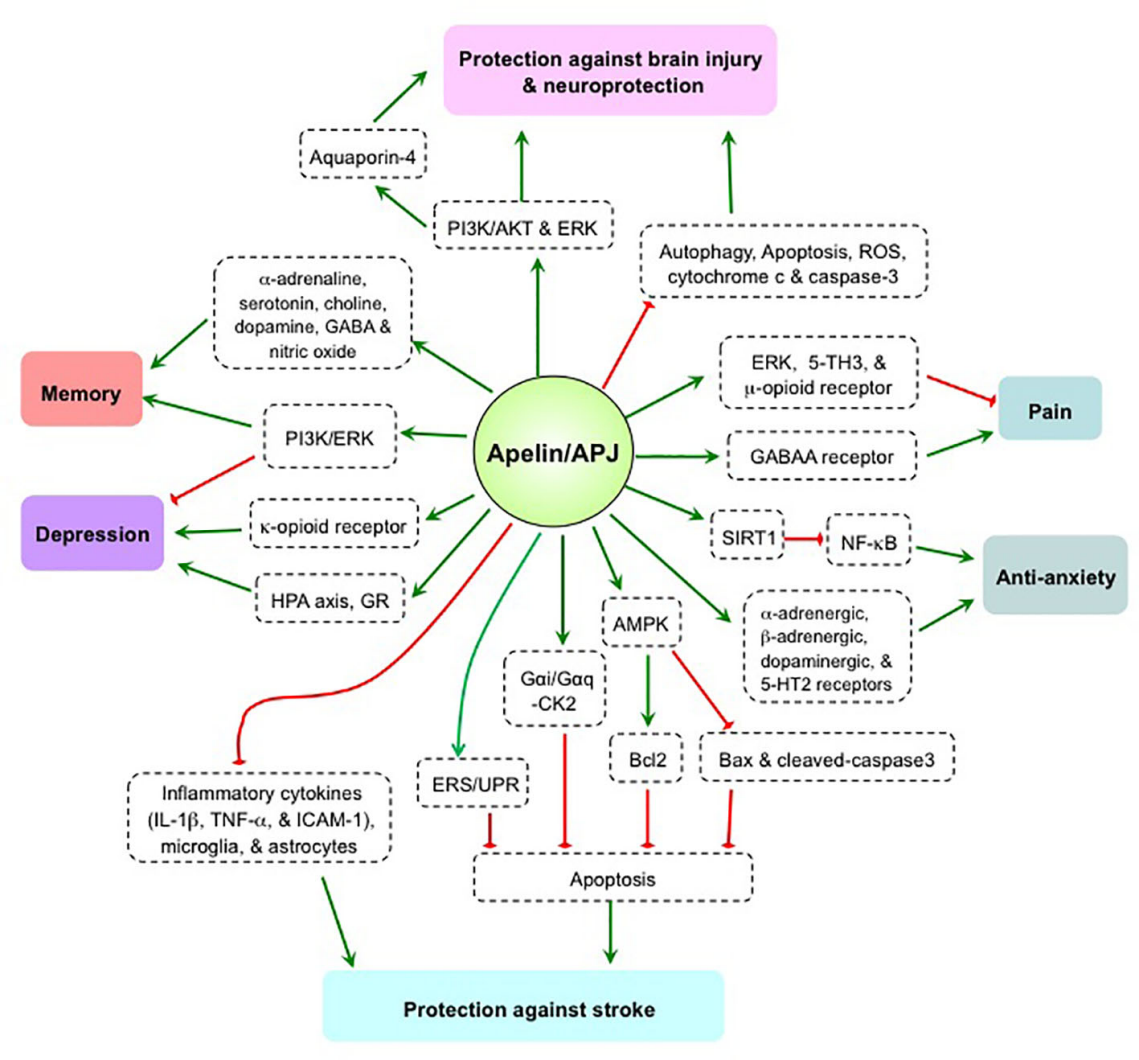
The mechanism and effect of apelin/APJ system on pain, anxiety, depression, memory, stroke, and brain injury. 5TH, 5-hydroxytryptamine; AKT, protein kinase B; AMPK, AMP-activated protein kinase; Bcl2, B-cell lymphoma 2; Bax, bcl-2 associated x protein; CK2, casein kinase 2; ERK, extracellular signal-related kinase; ERS/UPR, endoplasmic reticulum stress/unfolded protein response; GABAA, γ-aminobutyric acid, type A; GR, glucocorticoid receptor; HPA, hypothalamic–pituitary–adrenal; IL-1β, Interleukin-1β; ICAM-1, intercellular adhesion molecule 1; NF-κB, nuclear transcription factor-κB; PI3K, phosphatidylinositol 3-kinase; ROS, reactive oxygen species; SIRT1, silent mating type information regulation 2 homolog 1; TNFα, tumor necrosis factor α. Green arrow denotes stimulation. Red arrow denotes suppression.

The apelin/APJ system is strongly expressed in the brain, and plays a bi-directional regulatory role in pain, depression, and memory (**Figure 1**). Varying results in human and animal studies, however, are likely due to differences in research subjects, drug treatment methods, and experimental protocols. As such, contrary conclusions remain to be further explored. The apelin/APJ system has been shown to exert effects against stroke, brain injury, and anxiety, thus producing a neuroprotective effect mostly from apelin-13 administration models. However, the roles of endogenous apelin in the CNS are still unclear. Elucidation of the underlying mechanism(s) and the roles of endogenous apelin/APJ system using gene knockout or shRNA-mediated knockdown technology are needed to confirm whether the apelin/APJ system is a viable target for the treatment of human psychosis and neuropathy.

## Author Contributions

S-YL wrote the manuscript. W-DC and Y-DW revised and edited the manuscript.

## Funding

This work is supported by the National Natural Science Foundation of China (Grant Nos. 81472232, 81970726, and 81270522), the Plan for Scientific Innovation Talent of Henan Province and Henan Provincial Natural Science Foundation (Grant No. 182300410323 and 182300410316), the Program for Science & Technology Innovation Talents in Universities of Henan Province (HASTIT, Grant No. 13HASTIT024) to W-DC; the National Natural Science Foundation of China (Grant No. 81600974 and No. 81971280), the Key Science and Technology Program of Henan Province in China (Grant No. 192102310080), the Key Scientific Research Program for Universities of Henan Province in China (Grant No. 17A310003), the Fundamental Research Funds of Henan University (Grant No. yqpy20170040), the Key Science and Technology Program of Kaifeng City in China (Grant Nos. 1803034 and 1903019), and the Scientific Research Foundation of Henan University (Grant No. 2015YBZR050) to S-YL; the National Natural Science Foundation of China (Grant No. 81672433, No. 81970551, and No. 81370537), the Fundamental Research Funds for the Central Universities and Research Projects on Biomedical Transformation of China-Japan Friendship Hospital (Grant No. PYBZ1803), and the Fundamental Research Funds for the Central Universities (Grant Nos. PYBZ1706) to Y-DW.

## Conflict of Interest

The authors declare that the research was conducted in the absence of any commercial or financial relationships that could be construed as a potential conflict of interest.
