# Supplementary material for: The Apelin/APJ System in Psychosis and Neuropathy
Source: Front Pharmacol. 2020 Mar 13;11:320. doi: 10.3389/fphar.2020.00320 (PMC7082832; doi:10.3389/fphar.2020.00320)
Supplement: Supplementary file 1 [file Image_1.pdf]

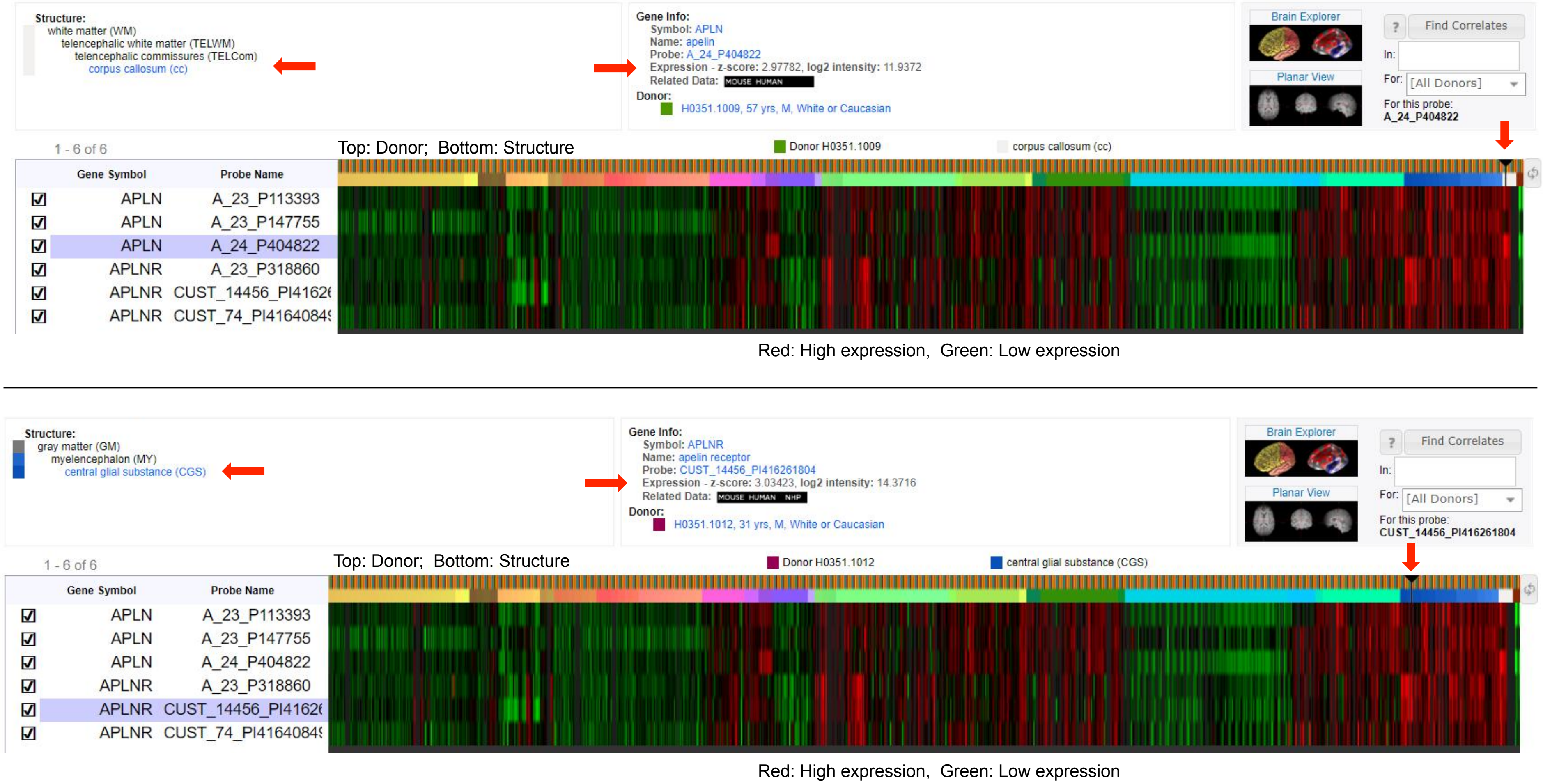

Supplementary Figure 1. The *APLN* and *APLNR* gene expression in different regions of the human brain. The data was analyzed and heat map was generated by Allen Human Brain Atlas (human.brain-map.org), Allen Institute for Brain Science. The number of donors was 3 for APLN and 3 for APLNR.
